# Towards the construction of health workforce metrics for Latin America and the Caribbean

**DOI:** 10.1186/1478-4491-9-24

**Published:** 2011-10-14

**Authors:** Gustavo G Nigenda, Maria H Machado, Fernando F Ruiz, Victor V Carrasco, Patricia P Moliné, Sabado S Girardi

**Affiliations:** 1 Health Services and Systems Innovations, Health Systems Research Centre, National Institute of Public Health, Cuernavaca, Mexico; 2 Senior Researcher. National School of Public Health. Osvaldo Cruz Foundation. Rio de Janeiro, Brazil; 3 Center for Project Development. Pontificia Universidad Javeriana, Bogota, Colombia; 4 Professor. Carlos Vidal Layseca School of Public Health and Administration. Cayetano Heredia Peruvian University. Lima, Peru; 5 Advisor. Plenitud Foundation, Santo Domingo, Dominican Republic; 6 Researcher. Center for Public Health Studies. Federal University of Minas Gerais. Belo Horizonte, Brazil

## Abstract

**Introduction:**

One of the components of the Health Observatory for Latin American and the Caribbean (HO-LAC) is the design and implementation of metrics for human resources for health. Under the HO-LAC initiative, researchers from nine countries in the region formed the Collaborative Community on Human Resources for Health in Latin America and the Caribbean to identify common metrics applicable to the field of human resources for health (HRH).

**Case description:**

The case description comprises three stages: a) the origins of an initiative in which a non-governmental organization brings together researchers involved in HRH policy in LAC, b) a literature search to identify initiatives to develop methods and metrics to assess the HRH field in the region, and c) subsequent discussions held by the group of researchers regarding the possibilities of identifying an appropriate set of metrics and indicators to assess HRH throughout the region.

**Discussion and evaluation:**

A total of 101 documents produced between 1985 and 2008 in the LAC region were identified. Thirty-three of the papers included a variety of measurements comprising counts, percentages, proportions, indicators, averages and metrics, but only 13 were able to fully describe the methods used to identify these metrics and indicators. Of the 33 articles with measurements, 47% addressed labor market issues, 25% were about working conditions, 23% were on HRH training and 5% addressed regulations. Based on these results, through iterative discussions, metrics were defined into three broad categories (training, labor market and working conditions) and available sources of information for their estimation were proposed. While only three of the countries have data on working conditions, all countries have sufficient data to measure at least one aspect of HRH training and the HRH labor market.

**Conclusions:**

Information gleaned from HRH metrics makes it possible to carry out comparisons on a determined experience in space and time, in a given country and/or region. The results should then constitute evidence for policy formulation and HRH planning and programs, with improved health system performance ultimately contributing to improved population health. The results of this study are expected to guide decision making by incentivizing the construction of metrics that provide information about HRH problems in LAC countries.

## Background

This paper describes the initial efforts of a regional non-governmental initiative in laying the foundation of a framework to analyse the field of human resources for health (HRH) in Latin America and the Caribbean. It provides information about the origins of the initiative, preliminary collection of information by means of a literature search and the ensuing discussions leading to a set of metrics and indicators to be used in monitoring HRH policies in the region.

Historically, as in other developing region,s the process of construction, dissemination, and utilization of information on HRH in Latin America and the Caribbean (LAC) has had its ups and downs. The past 10 years have seen uneven progress in the implementation of HRH information systems across countries in the region [1]. For many governments the issue of HRH is not a priority: on one hand planning and regulation are considered too complex due to the political nature underlying the issue; and on the other, investing in the creation of information systems is considered very expensive and offers no guarantee of providing expected benefits [2]. However, during the past decade a number of regional initiatives have emerged which encourage governments to consider the relevance of HRH and to promote and support the development of information systems.

In 1999 the Observatories of Human Resources in Health [3], an initiative led by the Pan American Health Organization (PAHO), were established with the participation of the International Labor Organization (ILO), the Economic Commission for Latin America and the Caribbean (ECLAC) and nine signatory countries: Brazil, Chile, Costa Rica, Ecuador, El Salvador, Jamaica, Mexico, Panama and Peru. By 2004, 21 countries in the Americas had joined the network of observatories [4]. Since then, more than a dozen international meetings have triggered numerous domestic and multinational activities, strategies, studies and collaborative projects capable of generating scientific information on the training, labor market and regulatory processes affecting technicians and professionals involved in health promotion and the provision of preventive and curative services for populations.

A 2005 meeting in Toronto was key, as representatives from governments, professional organizations and academic institutions from the Americas gathered together to identify strategic challenges. These included long-term policies to adjust HRH to health system changes and epidemiological profile dynamics, equitable geographical distribution of health workers, effective regulation of health workers, promotion of quality work environments for health workers and interaction mechanisms between training and employment institutions; all with the aim of improving the capacity of health care systems to achieve their goals. The meeting was convened by PAHO, the Canadian Ministry of Health and World Health Organization (WHO). Thirteen agreements were reached to achieve the aforementioned objectives, the most important being to consolidate the governance capacity of health and educational authorities and to increase investment in HRH development [5].

Despite these important developments, however, policies are still lacking key data that can define targets to be met by programmes and policies, monitor their development and allow evaluations to assess capacity to achieve these targets and goals. The availability of information is heterogeneous across the region, thus a comparative approach across countries has not been possible. Non-governmental initiatives geared toward the capacities and resources of health sector institutions have expanded in order to strengthen public policies. The case described in the following sections illustrates the relevance of non-governmental organizations in bringing together researchers and HRH specialists to discuss potential metrics and indicators to monitor country-based and regional policies. This effort, in turn, implies the possibility of harmonizing and amalgamating databases in order to be able to produce comparable metrics.

## Case description

### Initiative origins

The field of HRH in Latin America and the Caribbean reveals a broad and complex picture framed by the advancement of broad health systems reforms and the heterogeneous application of public policies to regulate and plan supply and demand of health personnel over time. Using various methodological approaches, the study of HRH in LAC in recent years is predominantly descriptive and mainly focused on aspects of training and the definition of educational content for different health disciplines.

Within this context, the Carso Health Institute, in collaboration with the Mexican Health Foundation, fostered the creation of a Mexico-based Health Observatory for Latin America and the Caribbean (HO-LAC), with one main component dedicated to the construction of metrics in human resources for health. Under this initiative the Collaborative Community on Human Resources for Health in Latin America and the Caribbean (COCORHS in Spanish) was established in 2008 in Mexico City. The network includes researchers from Argentina, Bolivia, Brazil, Chile, Colombia, Costa Rica, the Dominican Republic, Mexico, and Peru. Its main objectives are:

• To create a space for developing and promoting health metrics that generate useful information and evidence for decision making.

• To identify common metrics applicable to different areas of HRH in LAC, including the priority areas of training, labor market and working conditions, as well as regulatory issues.

To advance toward the achievement of these objectives, the COCORHS carried out, in the first trimester of 2009, initial searches and analyses of bibliographic material that included measures developed in the previously-mentioned topic areas and that had utilized some sort of metric. The following sub-section describes the methodology and results of this literature review.

Clearly, there have been various attempts to generate data, indicators and measures to monitor HRH performance not only for training processes but also to inform the labor market and other sectors, with varying degrees of success [6]. It is also clear, according to WHO reports, that country databases are frequently structured under different arrangements, which makes it difficult to design indicators according to pre-defined parameters and, consequently, to ensure their comparability.

Compiling comparable HRH metrics for the region presents a great challenge given the total or partial lack of - and dispersion of - data on HRH in LAC countries. Acknowledging the main constraints to accessing and using detailed, classified and disaggregated quantitative information on various HRH aspects, researchers from different LAC countries have launched a joint effort to construct standardized metrics permitting the systematization, comparison and analysis of data to support national and regional policy proposals on specific HRH topics.

### Literature review

The literature review was carried out in several stages, with the first stage consisting of bibliographic searches for publications about HRH in Latin America and the Caribbean containing quantitative data and measures. A search of grey literature and policy documents not included in public databases constituted another stage, the objective or which was to thoroughly review and identify studies originating in LAC countries containing HRH data, methods and metrics.

For the COCORHS, metrics are conceived of as a set of calculations that allow the measurement of individual and population aspects of health [7]. To provide an interpretative framework for this search, metrics were defined as 'the measurement(s) used to determine the quantitative dimension of the occurrence of a phenomenon in the field of HRH'. A metric should preferably correspond with some, if not all, of the following characteristics: a) be based on a theoretical construct, b) use standardized parameters for measurement and comparability, c) link two or more variables, and d) aim to guide decision making.

### Data collection and analysis

Publications were chosen for exhaustive review if they met the following criteria:

• Available in English, Spanish and Portuguese; published between 1980 and 2008. Since the majority of documents came from 1985 and later, this year was defined as the beginning of the period.

• Containing quantitative measurement indicators on HRH training, labor market or working conditions.

Publications containing only qualitative information or that were exclusively theoretical approaches to the field of HRH were excluded.

Health and social sciences databases such as LILACS, SciELO, EBSCO, the WHO search engine, and the Virtual Health Library were searched. In order to narrow the information we used the following keywords in Spanish (and the corresponding words in Portuguese): *personal de salud, recursos humanos para la salud, disponibilidad de recursos humanos para la salud, métricas de recursos humanos para la salud, análisis situacional de recursos humanos para la salud, perfil de los recursos humanos para la salud, indicadores de recursos humanos para la salud, salarios de los trabajadores de la salud, tendencias de la fuerza laboral en salud, entrenamiento del personal de salud*; and in English: health workforce, human resources for health, availability of human resources for health, metrics for human resources for health, situational analysis of human resources for health, profile of human resources for health, human resources for health indicators, health worker salaries, health labor force trends, health personnel training.

Also, COCORHS members were asked to submit the most up-to-date quantitative data and information on HRH, particularly grey literature and official reports from their respective countries considering the study's three broad fields, to help ensure that the most relevant publications and information in the field of HRH metrics were included.

Systematization of the collected documents was divided into two phases. The objective of the first phase was to have an initial situational analysis of HRH metrics in the region, thus all available documents from LAC were included. The second phase sought to identify HRH metrics developed in other regions to be used as a reference when constructing common metrics for the LAC region (this phase is yet to be completed).

The preliminary findings of the literature search were collectively discussed during two in-person meetings by the members of the network, this activity being described further in the third sub-section. The initial discussion sought to identify whether the broad areas defined in the theoretical framework matched with those identified in the literature and to consider the need to include other areas. After the discussion of each broad area, specific metrics were identified. The initial number of identified metrics was larger than the final number included. The final set of metrics was obtained by considering the relevance of the metric and the data available to estimate it.

From the literature search, a total of 101 documents with information on human resources for health originating in the LAC region were compiled; 33 of these included some type of measurement in the human resources field. The publications came from the following countries: Argentina (1), Brazil (7), Colombia (2), Costa Rica (1), Cuba (1), Dominican Republic (1), Ecuador (1), Mexico (7), Nicaragua (2), Paraguay (1), Peru (3), Uruguay (1) as well as several international collaborations (5) involving Bolivia, Colombia, Ecuador, El Salvador, Mexico, Peru and Venezuela.

From the 33 included publications a variety of measurements were identified, including counts (number of doctors); percentages (distribution by sex, employment sector); proportions (number of nurses per doctor); indicators (percentage of increase of HRH between time periods); and averages (annual number of consultations given by doctor). Only some of these measurements fit the metrics definition.

To inform the systematization of HRH metrics, the included papers were also classified according to type of publication, with the largest proportions corresponding to indexed articles (27.3%) and official government documents (including HRH plans and reports) (21.2%) (see Table 1).

**Table 1:** Number, percentage and type of publications presenting some type of measurement

Type of publication	Number	Percentage
Indexed articles	9	27
Articles published in non-indexed journals	6	18
Book chapters	3	9
Published books	4	12
Official documents	7	22
Others	4	12
**Total**	**33**	**100**

**Figure 1:**
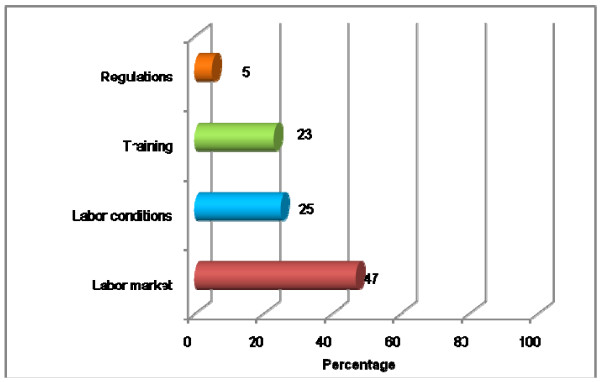
**Distribution of topics identified in the selected publications**.

Measurements included in the 33 publications addressed labor market issues (47%), working conditions (25%), HRH training (23%) and regulatory aspects (5%) (see Figure 1).

Based on the number of publications produced, prominent LAC countries include Mexico with 9 documents (27.2%), Brazil with 7 (21.2%), Peru with 3 (9.1%), and Bolivia and Colombia each with 2 (6.1% each). Of the total publications identified, 22% were the result of multi-country collaborations and/or working groups that develop international initiatives in the HRH field, as in the case of the OECD group.

More than three-quarters (24) of the publications included in the study used measurements such as ratios, proportions and percentages, while 21% (7 publications) contained more elaborately developed metrics that fit the previously outlined definition (Table 2).

**Table 2:** Publications with metrics descriptions.

Country	Metric
1. Brazil [18]	Develops an index to measure job instability among nursing personnel.

2. Colombia [19]	Proposes a formula for calculating HRH supply and demand. Quantifies tasks assigned to nurses and uses formulae to substitute occupational profiles.

3. Mexico [20]	Generates formulae to calculate labor wastage in five occupational categories.

4. Peru [21]	Presents information regarding the four areas of HRH and develops an analysis of in-country HRH distribution.

5. Peru [22]	Uses formulae for projecting physician demand and needs between 2006 and 2011. Makes projections of physician supply and needs in Peru.

6. Nicaragua [23]	Proposes formulae for calculating the number of physicians required according to hospital profile; and calculations to determine nursing personnel, general practitioners required to meet hospital admission demands, and nursing staff needs.

**7**. Mexico [24]	Uses formulae to measure occupational status, rates of unemployment, and underemployment.

## Discussion and evaluation

### COCORHS discussions to identify key metrics and indicators

This initial review of the literature yields results useful for building state of the art HRH metrics in Latin America and the Caribbean, identifying metrics on specific HRH issues, promoting comparative studies and formulating new goals in the creation of feasible and concrete metrics applicable to a large number of countries in the region.

All identified metrics have been selected with the aim that they reflect a specific dimension of human resources for health in the fields of training, employment, working conditions and regulatory aspects. As such, all metrics can be included in the conceptual framework which emphasizes the understanding of the HRH field as a market with specific types of regulations. It is possible to identify a larger number of metrics across these four fields. However, for the application of metrics to the design and monitoring of policies in developing countries, a certain level of pragmatism is important regarding the availability of data to be able to estimate them.

Moreover, as a central element in the discussion of metrics, HRH metrics should be capable of informing comparisons over time and space, in countries and/or regions, concerning a particular experience in the field of HRH [8]. In other words, metrics should permit comparisons that aim to identify the position of every country regarding a given issue. Some of the metrics identified have provided evidence for policy and program formulation and planning for human resources, particularly those related to training and geographical distribution. Developing short and medium term scenarios can be one of the most valuable applications of metrics for decision making in human resource planning [9].

Not only is current conceptual development in HRH metrics limited, but so too is the development of methods and formulae allowing the measurement of situations such as the relationships among health personnel, their working conditions and population health [10]. Developing HRH metrics related to the health status of the population requires deeper conceptual analysis and reflection, further complicating its applicability to different contexts.

Regarding the measurements in use in individual countries, one important advancement is that practically all of the countries have implemented systems to track the numbers of health workers and have estimated proportions of health personnel by such measures as population size, number of hospital beds, existence of formal employment ties, state or municipality, health worker level and/or area of training and whether a health worker has patient contact. It is now common to estimate the number and geographical distribution of HRH by area and/or type of health personnel, as well as existing institutional capacity to meet population health care needs and achieve national health goals. However, in health systems the development of human resources has been especially complicated by aspects such as increased demand, the ability or inability to respond to marked changes in a country's epidemiological profile, migratory trends and labor market dynamics [11].

The results of the literature review were discussed at two meetings held in San Jose, Costa Rica, in November of 2009 and in Rio de Janeiro in November 2010, attended by fifteen members of the COCORHS, representing eight countries in the network (Argentina, Brazil, Colombia, Costa Rica, Chile, the Dominican Republic, Mexico, and Peru) in addition to three international HRH experts.

Discussions held by the group in the Costa Rica meeting led to the following conclusions:

1. Three areas or categories were defined for developing and estimating HRH metrics: a) training, b) labor market, and c) working conditions. These areas match those previously defined by the theoretical framework. Regulatory issues were dropped due to a total lack of quantitative data available in the region to estimate them.

2. A total of seven topics within the three main areas were defined, two under in-training, three related to labor market and two under working conditions (Table 3). The second topic initially included in the labor conditions category was 'formal negotiation of employer-employee agreements', but was later changed to 'stable job'.

**Table 3:** Availability of information on specific topics as defined by COCORHS members, by category and country.

Categories and topics	Argentina	Brazil	Chile	Colombia	Mexico	Peru	**Dom. Rep**.
**TRAINING**							

Professional profile according to health delivery model		X	X	X	X		

Volume of professionals trained	X	X	X	X	X	X	X

**LABOR MARKET**							

Occupational status: employment, unemployment, under-employment, multi-employment		X		X	X		

Salary		X		X	X		X

Distribution: geographical, level of care, sex, sector (public-private)	X	X	X	X	X	X	X

**WORKING CONDITIONS**							

Workplace conditions	X		X		X		

Stable job	X	X			X		

The area with the fewest identified options for obtaining information was labor conditions, for which only Argentina, Brazil, Chile and Mexico have available data (Table 3). On the other hand, every country has the training and labor market categories covered by at least one of their defined metrics (Table 3). Additionally, all countries report having information on the volume of personnel trained and the distribution of HRH in the labor market. Regulatory mechanisms were not considered to be a field for metrics definition, as the vast majority of information in this area is not quantitative.

In Rio de Janeiro, follow-up discussions led to a still more simplified set of metrics due to data restrictions. For example, the original indicator 'labor wastage' summarizing unemployment and underemployment rates, has been estimated in Mexico for doctors [12] and nurses [13] but was dropped because there is no available data in other countries. A further agreement was also to estimate agreed metrics and indicators in each member country, an activity that is currently taking place. The group will eventually meet by the end of 2011 to discuss the findings of country cases.

### Discussion and evaluation of case study findings

The present document describes a strategy followed by a group of public health researchers in Latin America and the Caribbean to identify metrics in the field of human resources for health that could be used to monitor policies and inform decision makers on both country-specific and regional HRH issues.

The first step was to carry out a literature search to identify HRH metrics in line with a conceptual framework that included four broad topics and a specific definition of the metrics concept that led us to work with theoretically grounded measurements that could be comparable across countries and meaningful to the decision making process [14].

Representing only 7% of all the publications, those with specific metrics on the health workforce are quite scarce in the LAC region overall, with the exception of Brazil, Colombia, Mexico, Nicaragua and Peru, and the initiatives of international collaborations.

Of the metrics in HRH, 70% are concentrated in the fields of labor market and health personnel working conditions. In these areas, metrics typically are related to the estimation of employment, unemployment, unstable employment, underemployment, allocation of personnel, the potential demand for HRH and labor wastage. Surprisingly, scarce literature was identified in the area of training. Most publications addressing the topics of interest focus exclusively on doctors, nurses and dentists; rarely are other health sector professions included.

To arrive at a comprehensive situational analysis of the status of HRH training and the labor market, and the need to expand and/or reconsider regulations in either area, progress must be made in constructing systematic measurements with common methodologies at the local, national and international level. A comprehensive and detailed situational analysis on HRH is possible through creating and developing metrics. Efforts made during the past two decades in LAC have been important, but insufficient to generate macro and micro situational analyses by country or region. New indicators and metrics are needed to define and guide policy formulation and decision making.

The next step in the field of HRH is to promote similar research that includes a more extensive bibliographic review from various sources in different LAC countries, permitting a more intensive search for available metrics in additional areas in the HRH field, such as regulation, working conditions and workers' health. To enrich primary sources and expand the references in this field, it is of great importance to identify available databases within each country, specifying available quantitative HRH information with which metrics can be constructed.

Promoting metrics innovation and development is a rarely addressed area of HRH. Metrics can promote a deeper understanding of each of the topic areas and provide key information allowing policy tracking and monitoring. This will help to position HRH metrics as a fundamental tool for concrete aspects of planning, policy definition and decision making at the institutional and sector-specific level.

The results from this study can serve as a foundation from which to follow up, analyze and discuss the HRH metrics identified, with the goal of selecting some of the metrics for application in countries with a COCORHS representative.

This study's results will surely serve as an incentive for participating COCORHS countries to construct key metrics in each of the main HRH areas, in response to each country's priority problems, such that information can be generated and made available to decision makers. In fact, this will be the substantive contribution of the Collaborative Community's second phase of work. It is clear that the development of metrics has to consider cross-country comparison despite the existing differences in data production in each country. A first effort has to focus on standardizing the production of every metric, guaranteeing that available sources (particularly secondary ones) present data in a way that allows for the production of every selected metric, particularly as relates to coding and data aggregation.

Selection of metrics by COCORHS is based on the assumption that HRH participation is inter-linked across four broad fields. The first of them is the training provided by schools, through which the supply of individuals with specific capacities and skills is generated. These individuals eventually perform different activities related to the production of health services. These activities could be related directly to production by participation in clinical activities, or indirectly related through teaching, planning or evaluation of health provision.

The second field, labor market, generates the demand for those individuals trained at schools to be incorporated into the production process. However, generally speaking not all graduates can be incorporated into the health labor market, producing negative effects such as unemployment and underemployment.

The third field consists of the employment conditions of health personnel, which are mainly characterized by the type of link the personnel have with the labor market. The link can be salaried in cases when workers obtain a position within public and/or private institutions through a contract or verbal agreement. A second type of link is independent practice where a worker (mainly doctors and dentists) can set up their own practice and charge fees for services to their clients, or be paid by third party payers.

These three fields, in turn, are subjected to regulatory processes (the fourth field) which define, on the training side: the contents of training, duration, requisites for obtaining a degree, training at specialty levels, etc. On the labor market side regulations define: aspects of planning and geographical and institutional distribution. For working conditions, issues such as type of contract, work schedule, income and benefits are considered. It is also important to point out that regulatory agencies are normally different from those which train and contract personnel. Metrics and indicators selected by COCORHS can be located in these four fields [15,16].

## Conclusions

An HRH metric should provide information permitting assessments over time, and in a specific country or region, with regard to a given phenomenon in the HRH field. Furthermore, the metric should guarantee comparability, with the goal of identifying phenomena that can be expressed numerically. Information provided by metrics should constitute solid evidence within the tasks of formulating policies, plans and programmes for HRH and with the ultimate goal of improving the performance of health systems as a means to improve population health conditions.

One of the most valuable areas for metrics application is the HRH field, as it could be useful in carrying out planning of HRH within the health care system, framed in a manner that allows policy makers to make adequate decisions for short and long term adjustments.

Laying a foundation for the selection and estimation of metrics is difficult due to the lack of a conceptual framework. So, too, is the development of methods and metrics that could allow the measurement of phenomena concerning the link between health personnel, their participation in health activities, and population health indicators. The development of metrics linking HRH with health conditions requires deeper analytical and conceptual developments, making this a later analytical stage [17].

Regarding measurements developed across countries, an important advancement is the fact that most countries (including developing ones) have put in place systems to count health personnel and identify indicators related to population size, contact with patients, number of beds and health care units. It may be a common denominator today to be able to estimate the presence of health personnel by region and the institutional capacity to provide health services to populations and reach health goals. However, the participation of HRH within health systems has become more complex due to elements related to organizational complexities, the capacity to respond to an evident change in the population's epidemiological profile and labor market dynamics. Innovations are needed to respond to most of these challenges, including adequate performance of HRH within institutions.

The lack of academic and policy documents that propose specific HRH participation metrics for HRH is evident, with exceptions identified in Bolivia, Brazil, Colombia, Mexico, Nicaragua and Peru, as well as the initiatives developed by international agencies. Metrics are concentrated in educational and labor market issues, but scarce in working condition-related and regulatory issues.

It is of critical importance to discuss theoretical frameworks that could provide the foundation for relevant metrics in the HRH field. Although metrics have been described for specific LAC countries it is important to homogenize these estimations throughout the region and make them comparable. In some countries, data sources are available but not used for these purposes, therefore it is of the essence for researchers to identify and use them. Further efforts are needed to develop and apply relevant metrics with the participation of all interested actors, governments, researchers, NGOs and international agencies.

## Competing interests

The authors declare that they have no competing interests.

## Authors' contributions

GN coordinated the bibliographic review and the systematization of the information collected and was responsible for the study design and analysis. MM participated in the development of the theoretical framework and the final analysis. FR participated in the design of the analytical strategy. VC participated in the literature review, and PM and SG participated in the analysis of final information. All authors have read and approved the final manuscript.

## Authors' information

GN, MM, and SG, have carried out research in the HRH field for the past 20 years as well as in the health policy field. VC and FR teach health topics in prestigious private universities in Peru and Colombia respectively, including issues in HRH. PM participates in civil society organizations in the Dominican Republic and other countries, carrying out evaluations of social programs.

## References

[B1] PAHO Health human resources trends in the Americas: evidence for action 2006 Washington, D.C: PAHO

[B2] Novick M Desafíos de la Gestión de los Recursos Humanos en Salud: 2005-2015 2006 Washington, D.C: OPS

[B3] Organización Panamericana de la Salud Observatorio de los Recursos Humanos en Salud 2000 Quito

[B4] Observatorio de Recursos Humanos en salud en Brasil http://www.opas.org.br/rh/publicacoes/textos/ACF398.pdf

[B5] OPS/OMS: Llamado a la Acción de Toronto 2006-2015 Hacia una Década de Recursos Humanos para la Salud en las Américas. Reunión Regional de los Observatorios de Recursos Humanos en Salud: 4 al 7 Octubre 2005 2006 Ontario, Canadá. Editado por OPS/OMS 1 9

[B6] Bloor K Maynard A Planning Human resources in health care: Towards an economic approach An International comparative review. Ottawa Canadá 2003

[B7] Metricas de la Salud http://www.sinais.salud.gob.mx/metrica/index.html

[B8] Anand S Bärnighausen T Health workers and vaccination coverage in developing countries: an econometric analysis Lancet 2007 369 1277 85 10.1016/S0140-6736(07)60599-6 17434403

[B9] Dussault G Dubois CA Human resources for health policies: a critical component in health policies Human Resources for Health 2003 1 1 10.1186/1478-4491-1-1 12904254 PMC166115

[B10] Anand S Bärnighausen T Human resources and health outcomes: cross-country econometric study Lancet 2007 369 1277 1285 10.1016/S0140-6736(07)60599-6 15519630

[B11] Simoens S Villeneuve M Hurst J The Supply of Physician Services in OECD Countries 2003 OECD Health Working papers NO. 21. Paris

[B12] Nigenda G Ruiz JA Bejarano R Educational and labor wastage of doctors in Mexico: towards the construction of a common methodology Human Resources for Health 2005 3 3 10.1186/1478-4491-3-3 15833105 PMC1087866

[B13] Nigenda G Ruiz JA Rosales Y Bejarano R Enfermeras con licenciatura en México: estimación de los niveles de deserción escolar y desperdicio laboral Salud Pública Méx 2006 48 22 29 16555531 10.1590/s0036-36342006000100005

[B14] Diallo K Zurn P Gupta N Dal Poz MR Monitoring and evaluation of human resources for health: an international perspective Human Resources for Health 2003 1 3 10.1186/1478-4491-1-3 12904252 PMC179874

[B15] WHO Colaboremos por la salud. Informe sobre la salud en el mundo 2006 WHO. Ginebra 135 152

[B16] Nigenda G Ruiz JA González LM Wirtz V González MC Aguilar E Bejarano R Formación, empleo y regulación de los recursos humanos para la salud: Bases para su planeación estratégica 2010 Instituto Nacional de Salud Pública/Centro de proyectos para el desarrollo Universidad Pontificia Javeriana. Colombia ISBN: 978-958-716-397-1.

[B17] Development of Regional HRH Indicators and Monitoring Template http://www.aaahrh.org/documents/draft_monitoring.pdf

[B18] Feitosa J De Freitas J Meneleu J Precarización del trabajo de nivel técnico en salud en el nordeste: un enfoque en las categorías de auxiliar y técnico de enfermería 2004 OPS/OMS

[B19] Ruiz F Camacho S Eslava J Recursos Humanos de Salud de Colombia. Balance, competencias y prospectiva 2007 Ministerio de la Protección Social/Pontificia Universidad Javeriana

[B20] Nigenda G Ruiz JA González LM Wirtz V González MC Aguilar E Bejarano R Formación, empleo y regulación de los recursos humanos para la salud: Bases para su planeación estratégica 2010 Instituto Nacional de Salud Pública/Centro de proyectos para el desarrollo Universidad Pontificia Javeriana. Colombia ISBN: 978-958-716-397-1.

[B21] Arroyo J Los sistemas descentrados de recursos humanos en salud: el caso del Perú, 1990-2005 2005 Universidad Peruana Cayetano Heredia

[B22] Carrasco V Lozano E y Velásquez E Análisis actual y prospectivo de la oferta, demanda y necesidad de médicos en el Perú 2005 2007 Consejo Nacional del Colegio Médico del Perú

[B23] Ministerio de Salud de Nicaragua Manual de procedimientos de planificación y programación de recursos 2007 Managua20385412

[B24] Frenk J Robledo C Nigenda G Patrones de empleo médico en las áreas urbanas de México Psicología y Salud 1988 1 31 3515006166

